# Ectopic localization of CYP11B1 and CYP11B2-expressing cells in the normal human adrenal gland

**DOI:** 10.1371/journal.pone.0279682

**Published:** 2022-12-30

**Authors:** Céline Duparc, Paméla Camponova, Malanie Roy, Hervé Lefebvre, Michaël Thomas

**Affiliations:** Department of Endocrinology, INSERM, NORDIC UMR 1239, CHU Rouen, Univ Rouen Normandie, Rouen, France; University of Colorado Boulder, UNITED STATES

## Abstract

The sharp line of demarcation between zona glomerulosa (ZG) and zona fasciculata (ZF) has been recently challenged suggesting that this interface is no longer a compartment boundary. We have used immunohistochemical analyses to study the steroid 11β-hydroxylase (CYP11B1) and aldosterone synthase (CYP11B2) pattern of expression and investigate the remodeling of the adrenal cortex in relation to aging. We analyzed human adrenal glands prepared from 47 kidney donors. No aldosterone-producing micronodules (APMs) were detectable in the younger donors aged between 22–39 but the functional ZG depicted by positive CYP11B2 staining demonstrated a lack of continuity. In contrast, the development of APMs was found in samples from individuals aged 40–70. Importantly, the progressive replacement of CYP11B2-expressing cells in the histological ZG by CYP11B1-expressing cells highlights the remodeling capacity of the adrenal cortex. In 70% of our samples, immunofluorescence studies revealed the presence of isolated or clusters of CYP11B2 positive cells in the ZF and zona reticularis. Our data emphasize that mineralocorticoid- and glucocorticoid-producing cells are distributed throughout the cortex and the medulla making the determination of the functional status of a cell or group of cells a unique tool in deciphering the changes occurring in adrenal gland particularly during aging. They also suggest that, in humans, steroidogenic cell phenotype defined by function is a stable feature and thus, the functional zonation might be not solely maintained by cell lineage conversion/migration.

## Introduction

The zonation of the human adrenal cortex has been established on morphological characteristics and arrangement of the constitutive cells [1, 2]. The names of the three zones are the result of their histological description. Thus, the zona glomerulosa (ZG) is made of cells arranged in baskets that resemble the glomeruli of the kidney. The cells of the zona fasciculata (ZF) lie in parallel radial columns or fascicles centripetally oriented. Finally, the innermost layer of the cortex is the zona reticularis (ZR), where the cells are organized in a network. It was only several decades later that the function and the identity of steroid product of each zone were discovered. Mineralocorticoids were found to be produced by the ZG cells; glucocorticoids by the ZF cells; and weak androgens by the ZR cells [3–5]. Especially, the enzyme responsible for the last steps of aldosterone biosynthesis, aldosterone synthase (CYP11B2), is only expressed in the histological ZG, while 11β-hydroxylase (CYP11B1), which catalyzes the last reaction of cortisol production, is expressed in the histological ZF and ZR [6]. The availability of specific antibodies against mice and rats CYP11B1 and CYP11B2 has reinforced the concept that the functional zonation follows the histological zonation [7–9] with a clear delineation between expression of CYP11B2 in ZG and CYP11B1 in ZF. The direct translation of the existence of a sharp boundary between zones in the human adrenal cortex was generally accepted due mainly to the lack of immunological tools for the detection and localization of the two CYP11B enzymes. However, Gomez-Sanchez et al [10] have succeeded in developing specific antibodies against human CYP11B1 and CYP11B2, allowing reappraisal of cortex zonation. With the use of these antibodies it appeared that the normal adult human adrenal cortex zonation was more complex than in rodents. The expression of CYP11B2 was mostly continuous in adrenal glands derived from young individuals, whereas its expression became discontinuous and sparse in adrenal glands older subjects [10–12], with the exception of rare clusters of CYP11B2 expressing cells, called aldosterone-producing cell clusters (APCCs). These APCCs have initially been defined as groups of morphological ZG cells close to the capsule and inner ZF-like cells isolated from the neighboring CYP11B1 expressing cells by a defined border [6, 11]. Recently, an international consensus recommended using the term aldosterone-producing micronudules (APMs) to define these structures [13]. In addition, subcapsular steroidogenic cells that do not exhibit CYP11B2 immunoreactivity were found to express CYP11B1 [10]. Collectively, these data indicate that assignment of an adrenocortical cell to a specific histological zone is not strictly predictive of its function. Therefore, it appears that reexamination of the zonation of the human adrenal cortex is essential for a better understanding of the normal adrenal gland biology. Herein, we have questioned the relevance of specific markers of the ZG and determined the Ki-67 proliferation index in CYP11B1- and CYP11B2-expressing cells.

## Materials and methods

### Normal human adrenal samples

Human adrenal glands were obtained from brain-dead donors without overt hypertension or long-term steroids use at the University Hospital of Rouen (S1 Table). After removal of the periadrenal fat tissue, each gland was cut perpendicularly to the longitudinal axis. Paraformaldehyde-fixed paraffin-embedded tissue blocks from 47 adrenals (collected from 18 female (38%) and 29 male (62%) patients, ranging in age from 22 to 81 with a median age of 54 without overt pathological findings were used for the study.

### Histological and immunohistochemical (IHC) analyses

Each adrenal was cut to a thickness of 5 μm. The slides were de-paraffinized and subjected to heat-induced epitope retrieval using either Tris-EDTA buffer pH 9 or Sodium Citrate buffer pH 6 for 10 to 25 min according to the antibody in use. After peroxidase blocking, the slides were saturated using TBS-BSA 2%, 5% normal goat serum for 20 min and then subsequently incubated at room temperature for 60 min with primary antibodies as follows: CYP11B2 (clone 41-17B, either a gift from Dr Gomez-Sanchez or purchased from Millipore; 1:500), CYP11B1 (clone 80–7, either a gift from Dr Gomez-Sanchez or purchased from Millipore; 1:400), HSD3β2 (clone 6, a gift from Dr Gomez-Sanchez, 1:5000), CYP17A1 (clone 10/19-G6-7-10, a gift from Dr Gomez-Sanchez, 1/3000), Ki-67 (clone MIB-1, Dako; 1:100), SF-1 (clone EPR19744, Abcam; 1/1000), Dab2 (clone H-100, Santa Cruz Technology; 1:500), β-catenin (clone 14, BD Transduction Laboratories; 1:400), Lef-1 (clone EPR2029Y, Abcam; 1:100), KCNJ5 (clone 36-33-5, a gift from Dr Gomez-Sanchez; 1:400) and Tyrosine hydroxylase (Sigma-Aldrich, 1:500) were applied in TBS-BSA 2%. The slides were then incubated with either an anti-mouse En Vision+ System-HRP (Dako), or an anti-rabbit En Vision+ System-HRP (Dako) or an anti-rat-HRP (Neo Biotech, Nanterre, France) according to the primary antibody in use. Slides were developed with diaminobenzidine and counterstained with hematoxylin. Consecutive sections were also prepared for H&E and CYP11B2 IHC staining, and CYP11B2 and Ki-67 IHC staining.

The slides were then photographed using a Nikon Eclipse E600 Microscope equipped with a Sony DXC-390 3-CCD Color Camera. All images were obtained on Primacen, the Cell Imaging Platform of Normandie, University of Rouen Normandie.

### Quantification of CYP11B2 expression

Images of CYP11B2 and SF-1 immunohistochemistry for each adrenal were captured using a binocular Zeiss Axioscope 7 coupled with an Axio Cam ICc5 CCD color video camera (Zeiss). Two to ten images per section depending on the size of the tissue were seized at a resolution of 2452 x 2056 and saved as TIFF files. The composite digital pictures built from these images were then analyzed using ImageJ software. The immunostained section for SF-1 allowed the determination of the total surface of the adrenal gland. A thresholding is then performed to define the areas occupied by the cortex and the medulla. The threshold tool was used from the CYP11B2 immunostained section to measure the surface areas expressing CYP11B2. The CYP11B2 positive surface/adrenal cortex surface was then calculated.

### Double immunofluorescence (IF) analysis

Adrenal slices were incubated for 60 min with a mixture of antibodies (rat anti-human CYP11B1 1/50, mouse anti-human CYP11B2 1/150, mouse anti-human Ki-67 1/100 and rabbit anti-rat Tyrosine Hydroxylase 1/500 as requested). After washing, a mixture of the following donkey made secondary antibodies was applied: anti-rat IgG Alexa Fluor 594 (A21209), anti-mouse IgG Alexa Fluor 488 (A21202), anti-mouse IgG Alexa Fluor 594 (A21203) and anti-rabbit IgG Alexa Fluor 488 (A21206) (all from Invitrogen) at a dilution 1:300 for 60 min. Sections were counterstained with DAPI at 1μg/ml for 10 min. Coverslips were mounted using Fluoromont (Sigma). For the KCNJ5 and CYP11B2 double IF, we used an anti-mouse IgG2b Alexa Fluor 594 secondary antibody (to detect KCNJ5 antibody, A21145) and an anti-mouse IgG1 Alexa Fluor 488 secondary antibody (to detect CYP11B2 antibody, A21121) diluted at 1/200 (Invitrogen).

### Evaluation of Ki-67 expression in CYP11B1- and CYP11B2-expressing cells

For each adrenal, consecutive immuno-stained sections for CYP11B2 and Ki-67 were analyzed. Four to six digital images for CYP11B2 and Ki-67 expressions were obtained from analogous location of each tissue section at x200-fold magnification. All images were printed in color at a size of 8 by 10 inches. The CYP11B2 positive area was delineated on the Ki-67 print and the proliferative cells expressing CYP11B2 were manually counted. To evaluate the Ki-67 expression in the CYP11B1 expressing cells, 4 to 6 merge double-label IF images for each adrenal were analyzed. Finally, the positivity index was obtained by calculating the percentage of cells with Ki-67 expressing cells among CYP11B1 and CYP11B2 expressing cells.

### Triple IF analysis

Triple IF was performed using antibodies for CYP11B1 and CYP11B2 and either Dab2, SF-1 or KCNJ5 antibodies. The samples were processed as above, with the exception that the 3 primary antibodies were added concurrently. After washing, a mixture of three secondary antibodies was used, including donkey anti-mouse IgG Alexa Fluor 488, donkey anti-rat IgG Alexa Fluor 594 and donkey anti-rabbit IgG Alexa Fluor 647 (FP-SC5110, Interchim, France) at a dilution of 1:300 for 60 min.

Immunofluorescent images were obtained using a TCS SP8 confocal laser-scanning microscope system (Leica Microsystems). All images were obtained on the Primacen platform.

### Statistical analysis

Statistical analysis has been performed by using Pearson correlation coefficients to analyze the association between age and CYP11B2 positive areas. Differential Ki-67 protein expression between CYP11B1- and CYP11B2- expressing cells was analyzed using Paired t-test, with a p value < 0.05 considered statistically significant.

### Study approval

The protocol of collection of the tissues and the experimental procedures were approved by the French Biomedicine Agency (Agence de Biomédecine, # PFS11-011). All research experiments were carried out in accordance with the guidelines and regulations of the Rouen Normandie University and INSERM. Written informed consents were obtained from all patients’ closest relatives.

## Results

**Fig 1:**
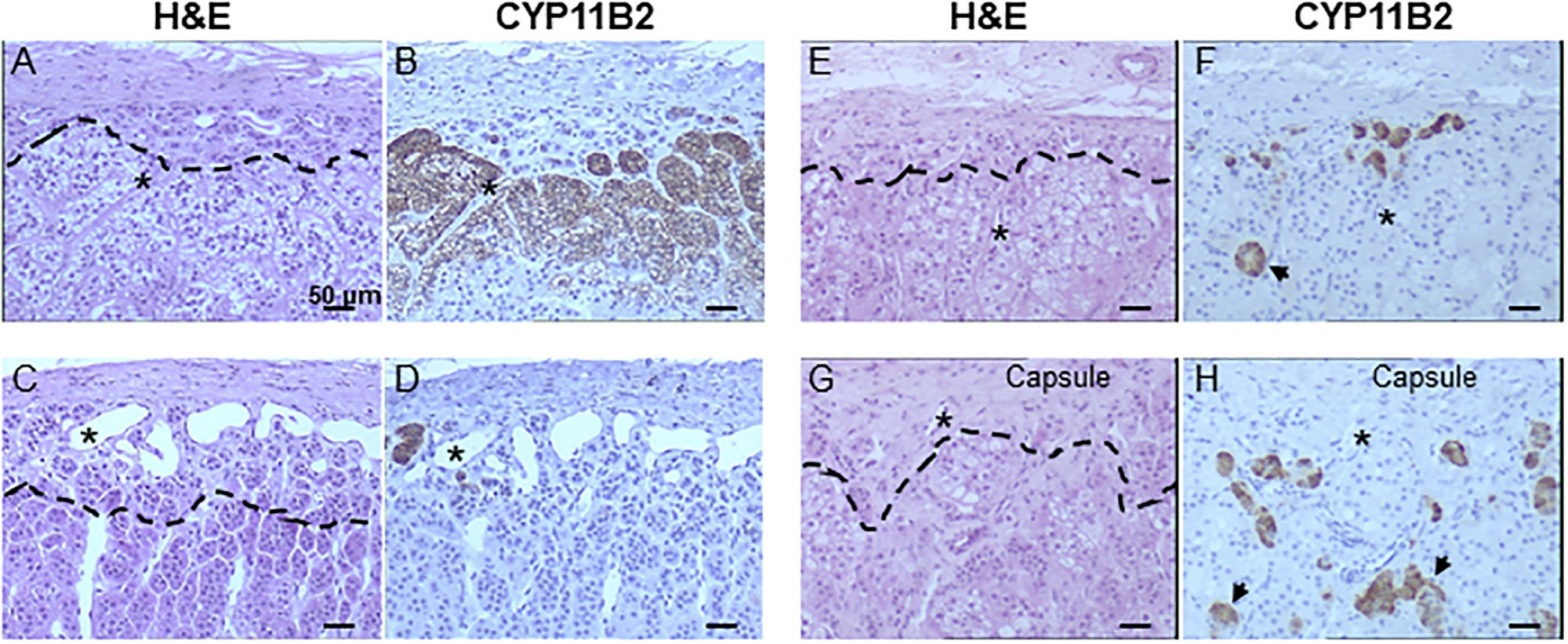
Comparison of histological and functional ZG. H&E staining and CYP11B2 immunolabelling (brown) of consecutive adrenal sections reveal a weak degree of superimposition of histological and functional ZG. 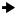 indicates CYP11B2 cell clusters migrating into the zona fasciculata. - - - delineates the histological boundary between zona glomerulosa (above) and zona fasciculata (below). (A-D) 22-year-old man. (E-H) 66-year-old man. The sections are matched in consecutive pairs, such as A-B and C-D for the 22-year-old and, E-F and G-H for the 66-year-old adrenal. * identifies common structures on consecutive sections.

We prepared two consecutive sections of each adrenal glands: the first was used for histological examination following H&E staining while the second was submitted to CYP11B2 immunohistochemical detection (Fig 1). Representative illustration of two different regions of a young (< 65 years) and an old (> 65 years) subjects is shown in Fig 1 (Panel A-D and Panel E-H). The compact cells histologically recognized as ZG cells do not all express CYP11B2- the ZG functional marker (Fig 1A–1D). In contrast, some cells with a large and clear cytoplasm, which could be histologically identified as ZF cells, expressed CYP11B2 (Fig 1B and 1F), confirming that the immunohistochemically-defined ZG cells were not consistent with the histological ZG defined by H&E staining. Interestingly, some clusters of these CYP11B2-positive ZF-like cells were not in contact with the capsule and consequently, could not be classified as APM (Fig 1B). Moreover, few CYP11B2 positive cells were remote from the subcapsular area (Fig 1F and 1H).

Representative CYP11B2 expression patterns in adrenal glands from different age are shown in S1 Fig. None of the 47 adrenals studied had a continuous CYP11B2 expression which appeared to be fragmented within the subcapsular region (S1A–S1F Fig). There was a strong reduction in the surface of CYP11B2 expressing area in the adrenal glands removed from older subjects, together with a thickening of remaining CYP11B2 expressing areas into clusters of CYP11B2 cells extending deeper into the cortex and defined as APMs (S1A–S1F Fig). Consistently with the previous report by Nanba et al (11), quantitative analysis of our data confirmed that the total area occupied by CYP11B2 expressing cells normalized to adrenal cortex area was negatively correlated with age (r = -0.3234, P = 0.0266, S1G Fig). Moreover, no APM was identified in the adrenal glands between the third and the fourth decades in our cohort while the decrease in the area occupied by the CYP11B2-expressing cells was already apparent.

**Fig 2:**
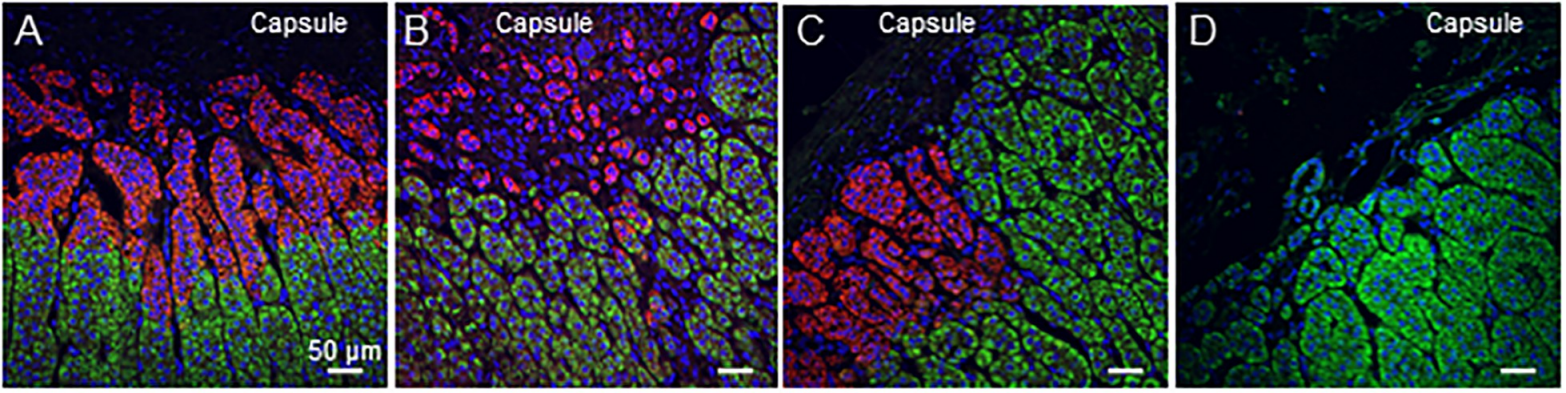
Co-immunofluorescence of CYP11B1 and CYP11B2 demonstrates adrenal remodelling. Illustrative merge photographs of CYP11B2 (red) and CYP11B1 (green) combined with nuclear staining with DAPI (blue) showing that the pattern of expression of the steroidogenic enzymes does not support the zonation defined by histological criteria in the adrenal cortex of a 68-year-old woman (A-D).

**Fig 3:**
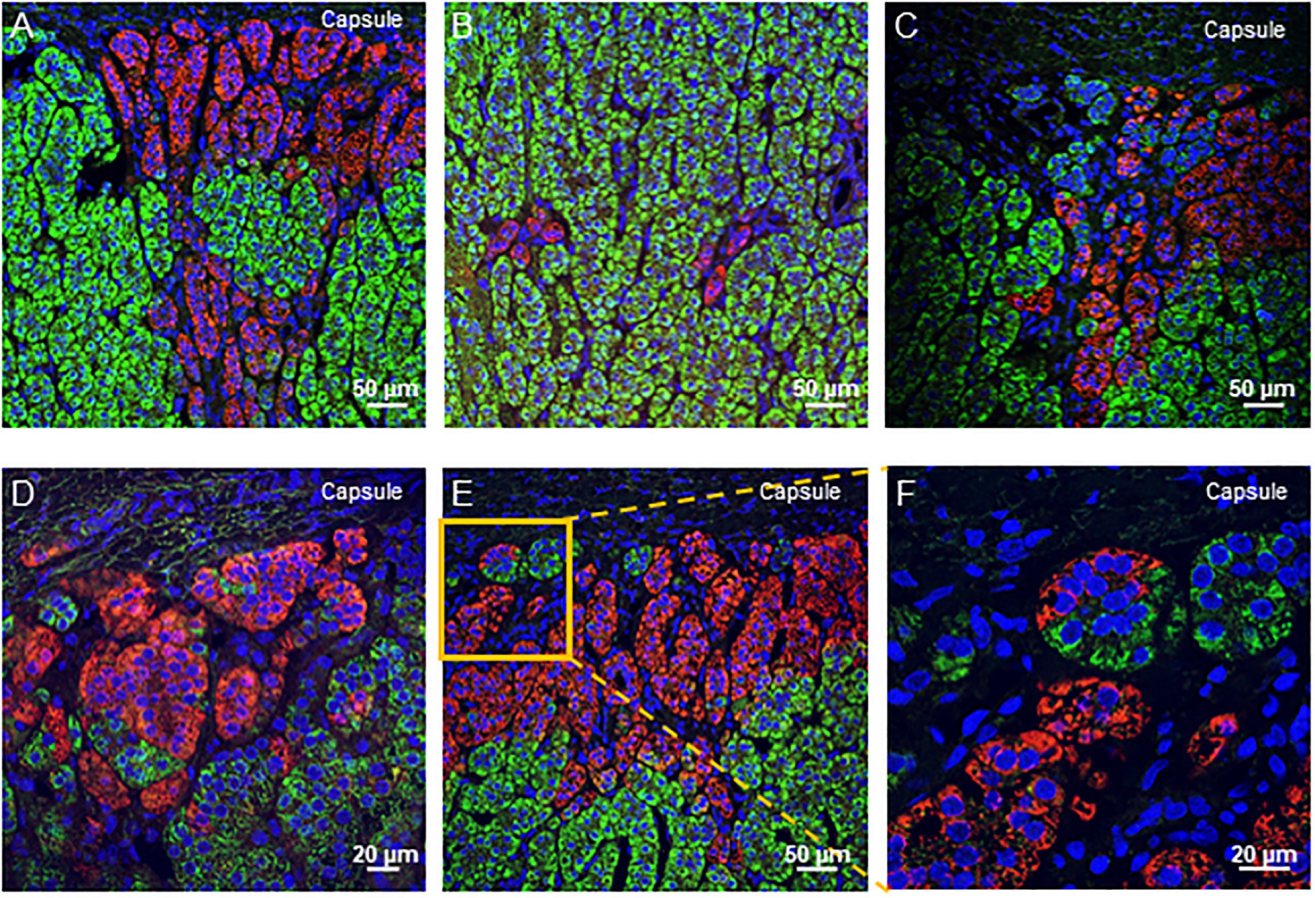
CYP11B1 and CYP11B2 positive cells are interwoven throughout the adrenal cortex. (A-F) Double immunofluorescence staining reveals the occurrence of CYP11B2 positive cells (red) in the ZF (CYP11B1 positive; green) in the adrenal cortex of a 49-year-old man. (A-B) In the inner cortex, clusters of CYP11B2 positive cells are intermingled with CYP11B1 expressing cells. (C-F) Both CYP11B1 and CYP11B2 positive cells participate in the formation of baskets found in the histological ZG.

Fig 2 shows overlapping immunofluorescence pictures of CYP11B1 and CYP11B2. Functional ZG cells which exclusively express CYP11B2 either formed small groups of cells just beneath the capsule or developed downwards in the subcapsular area to form the upper part of the cell cords that constitute the ZF without any defined boundary. This cell arrangement was found throughout our cohort (42/47, 89.36%) with no sex or age predominance (Fig 2A and 2B). The continuum between the CYP11B2 and the CYP11B1-expressing cells within these tissue structures precludes the recognition of CYP11B2-positive cells as APMs (Fig 2A). ZF cells which solely express CYP11B1, either formed columns reaching the outermost area of the cortex or small clusters beneath the capsule evoking glomerulosa cell baskets (45/47, 95.74% and 34/47, 72.34%, respectively, Fig 2C and 2D). It is noteworthy that the shape and the size of the CYP11B1- and CYP11B2-positive cell clusters located beneath the capsule were very similar (Fig 2B *vs*. 2D, 2A *vs*. 2C and 2D). Cells expressing respectively CYP11B1 and CYP11B2 were found interwoven at different levels: i) clusters of CYP11B2 cells can be observed remote from the capsule surrounded by CYP11B1-expressing cells (33/47, 70.21%, Fig 3A and 3B) and ii) the two type of cells might be found in the same nest or basket of cells beneath the capsule (40/47 donors, 85.10%, Fig 3A and 3D–3F). In order to authenticate the ectopic localization of CYP11B2 expressing cells in the cortex and their intertwining with CYP11B1-positive cells, consecutive sections were immunostained either for CYP11B2 or for CYP11B1 and CYP11B2. As shown in S4 Fig a cluster of CYP11B2 positive cells was first identified (section 0) after routine IHC. Subsequent sections revealed the entanglement of CYP11B2 expressing cells with CYP11B1 expressing cells with a progressive decrease in size of ectopic CYP11B2 cells that eventually disappeared (section 1 to 14). Interestingly, CYP11 B1 expressing cells can be trapped inside the group of CYP11B2 cells (section 7). Finally, these cells have no direct connection with the capsule nor contact with the medulla and thus seem to be intertwined within the cortex with CYP11B1 cells (S4 Fig). No cells were found to express both enzymes. However, it was conceivable that some cells might be negative for CYP11B1 and CYP11B2. We thus carried out a triple IF using Steroidogenic Factor 1 (SF1), a marker of corticosteroid producing cells. We observed no steroidogenic cell positive for SF1 and negative for both enzymes (S2 Fig).

**Fig 4:**
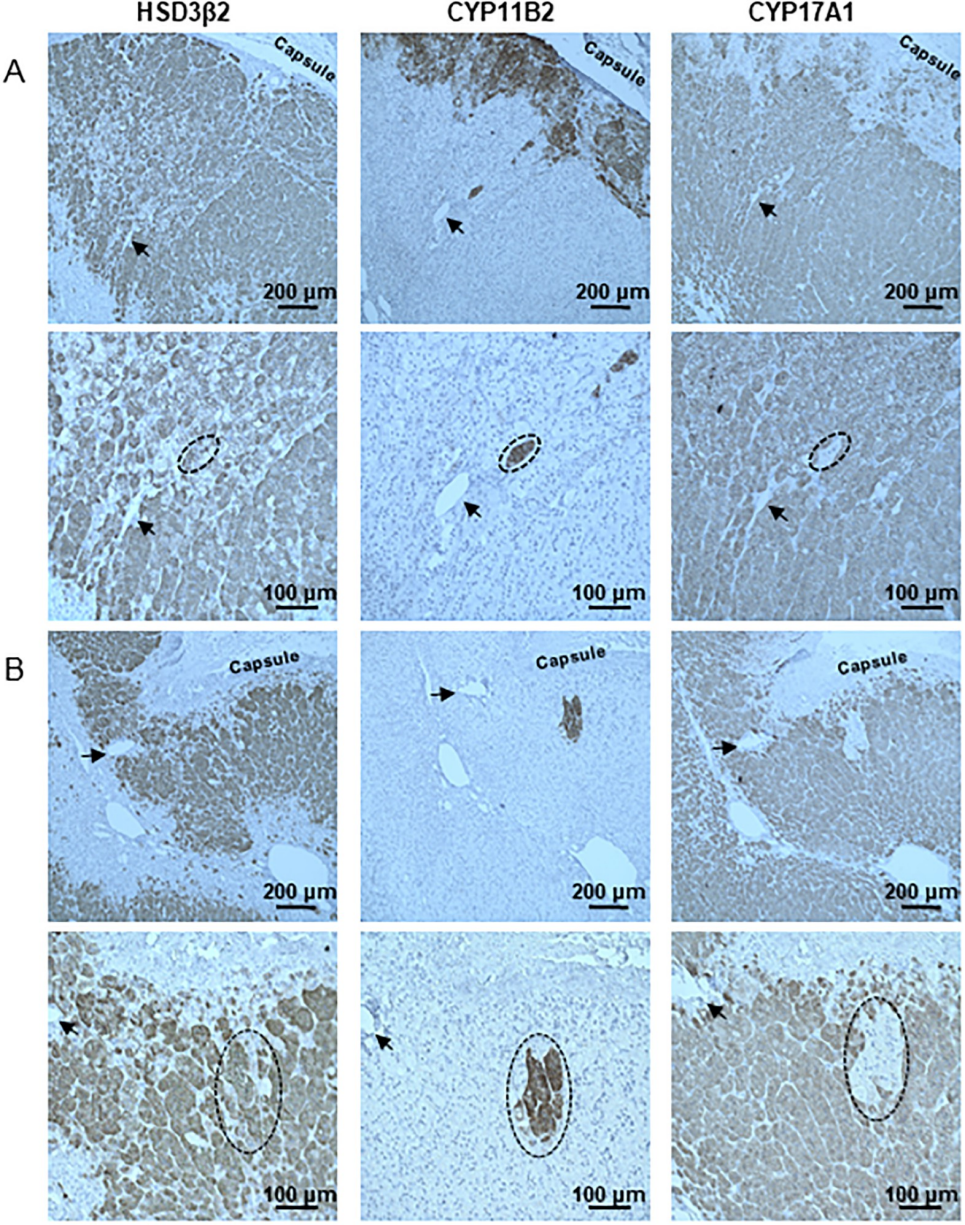
Phenotype stability of ectopically localized CYP11B2-positive cells. Expression of 3β-hydroxysteroid dehydrogenase type II (HSD3β2), CYP11B2, and 17α-hydroxylase (17CYPA1) in the adrenal gland from a 45-year-old woman (A) and a 66-year-old man (B).

To further define the phenotype of ectopically localized CYP11B2-expressing cells, 3 consecutive sections were cut from each adrenal. The first sections were labeled for 3β-hydroxysteroid dehydrogenase type II (HSD3β2), which is a marker of aldosterone- and cortisol-producing cells, the second sections were labeled for CYP11B2, and the last sections were labeled for 17α-hydroxylase (17CYPA1), which is a marker of cortisol- and androgen-producing cells. As shown, CYP11B2-positive cells express HSD3β2 but not 17CYPA1 (Fig 4A and 4B). These CYP11B2 cells thus retained the phenotypic characteristics that classify them as aldosterone-producing cells.

**Fig 5:**
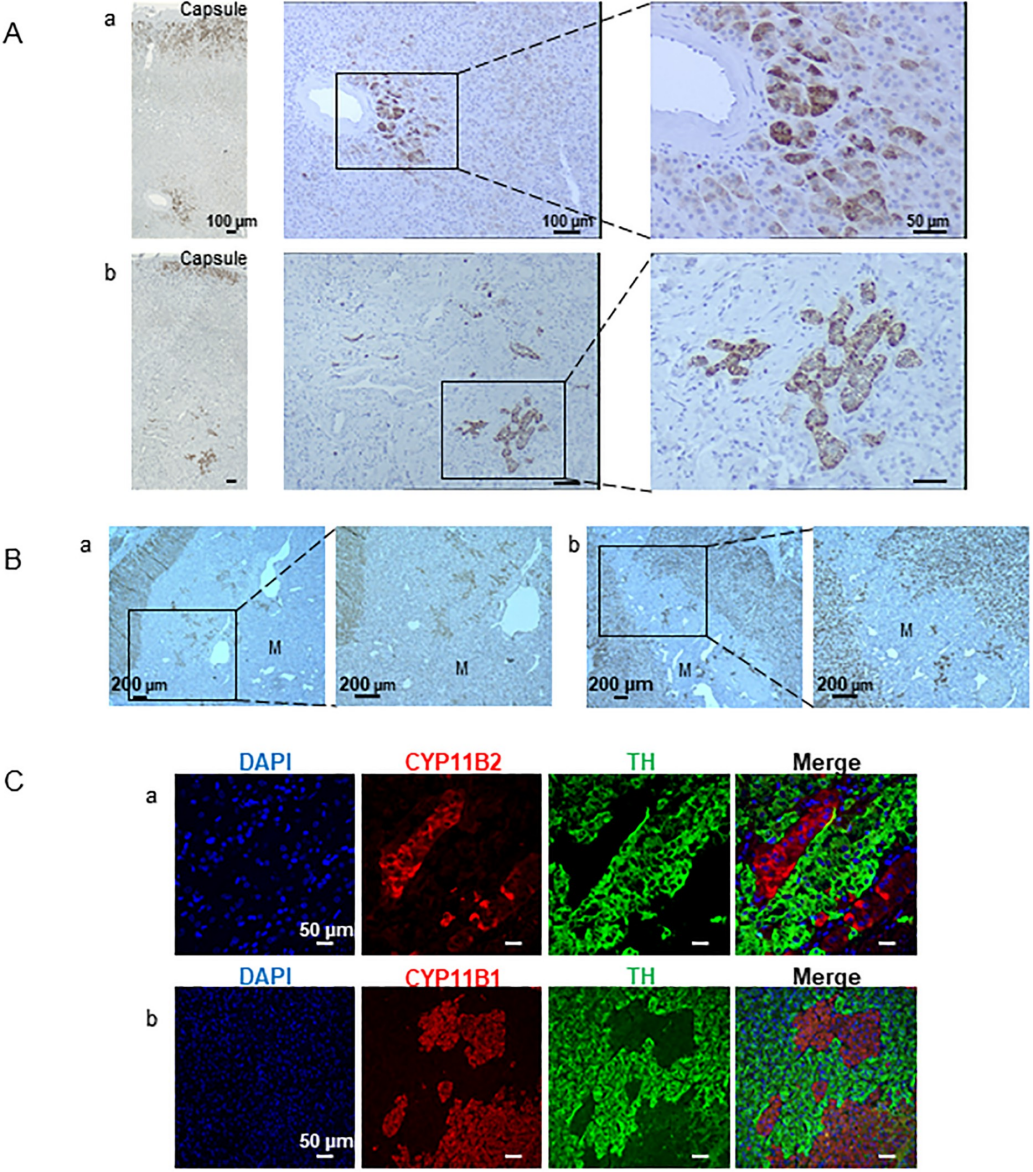
Ectopic localization of steroidogenic cells in the adrenal gland. (A) CYP11B2 immunostaining reveals that organized islets or isolated functional ZG cells can be identified in the vicinity of the central vasculature in the adrenal cortices of a 49-year-old man (a) and a 52-year-old woman (b). The left composite microphotographs show immunoreactivity for CYP11B2 throughout the cortex. (B) Immunostaining for SF1 highlights the occurrence of steroidogenic cells in the medulla (M). The adrenal medulla appears to be invaded by single or clusters of SF1-expressing cells. (a) 30-year-old man. (b) 72-year-old woman. (C) Double immunofluorescence for Tyrosine hydroxylase (TH) and either CYP11B2 or CYP11B1. The CYP11B2 (a) and CYP11B1 (b) positive cells are found within the adrenal medulla in close contact with the chromaffin cells. (a) 45-year-old man. (b) 64-year-old woman.

We also visualized CYP11B2 positive cells deep into the cortex and the medulla. These cells were unrelated to any APMs (Fig 5A). They appeared as single cells or arranged in small clusters of cells and ultimately, the cells were identified adjacent to a small artery or venule of the gland as shown in Fig 5A. Globally, we were able to identify CYP11B2 positive cells in various locations in the cortex besides their usual position beneath the capsule in 33 donors (70.21%): 11 (33%) were females and 22 (67%) were males and the presence of these cells was not age-dependent. As previously reported, examination of the corticomedullary junction showed that cortex and medulla were intermingled [14]. Clusters of cortical cells expressing SF1 were spread throughout the medulla and sometimes cortical cells lay around the medullary vessels surrounded by chromaffin cells (Fig 5B). 95.74% (45 out of 47) of the adrenals displayed more than 10 clusters of cortical cells into the medulla and 4.26% (2 out of 47) with 5 to 10 clusters (Fig 5B). Co-labelling of adrenal slices with antibodies against the chromaffin cell marker tyrosine hydroxylase and CYP11B1 or CYP11B2 showed that the medulla contains clusters of the two types of corticosteroid-producing cells (Fig 5C).

**Fig 6:**
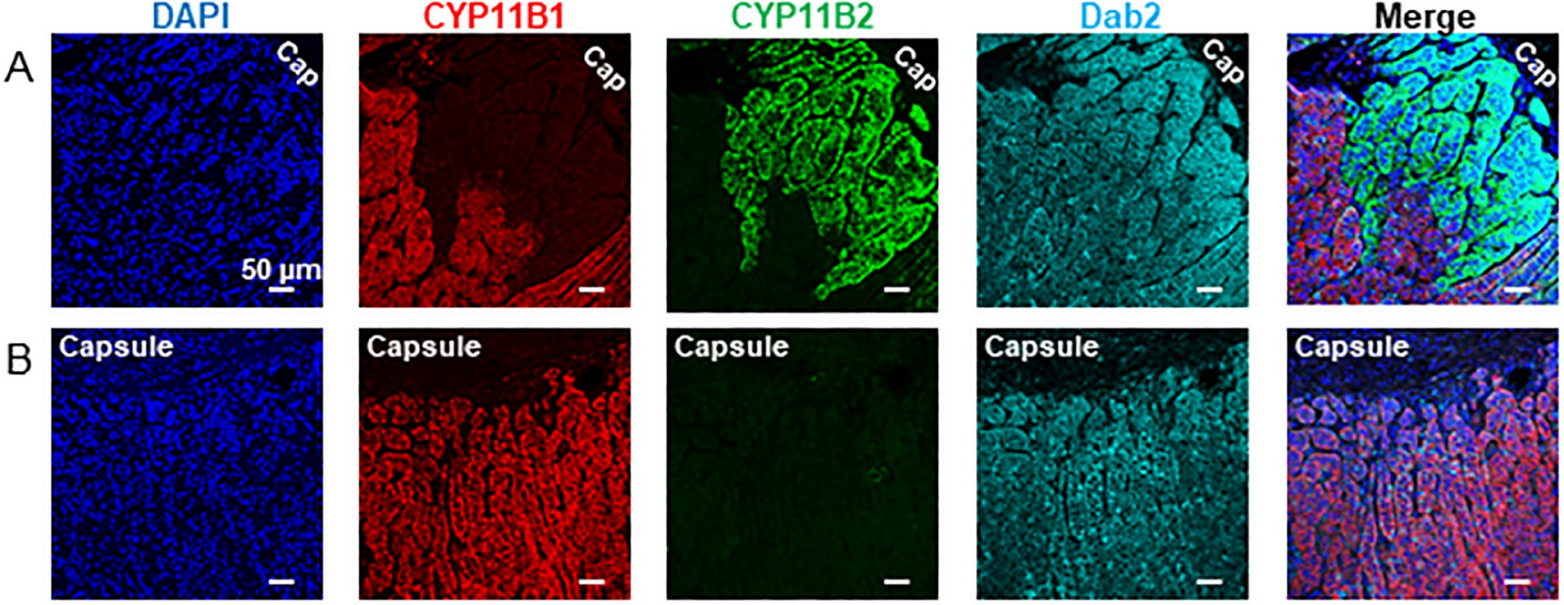
Dab2 expression is not restricted to the functional ZG. Triple immunofluorescence for CYP11B1, CYP11B2 and Dab2. Dab2 expression is observed throughout the adrenal cortex independently of the donor age and steroidogenic cell type. (A) 42-year-old man, Cap: Capsule. (B) 81-year-old man.

Disabled-2 (Dab2) has been shown to be a functional ZG specific marker in rats [15] and in humans [16]. In our collection of adrenal glands, the pattern of Dab2 expression was mostly membranous throughout the whole cortex, irrespective of donor age or sex (Fig 6A and 6B). Triple-staining IF studies indicated that Dab2 expression was not restricted to a particular cell type but was detected in all steroidogenic cells (Fig 6A and 6B) including the CYP11B2-positive cells constitutive of APMs (Fig 6A).

**Fig 7:**
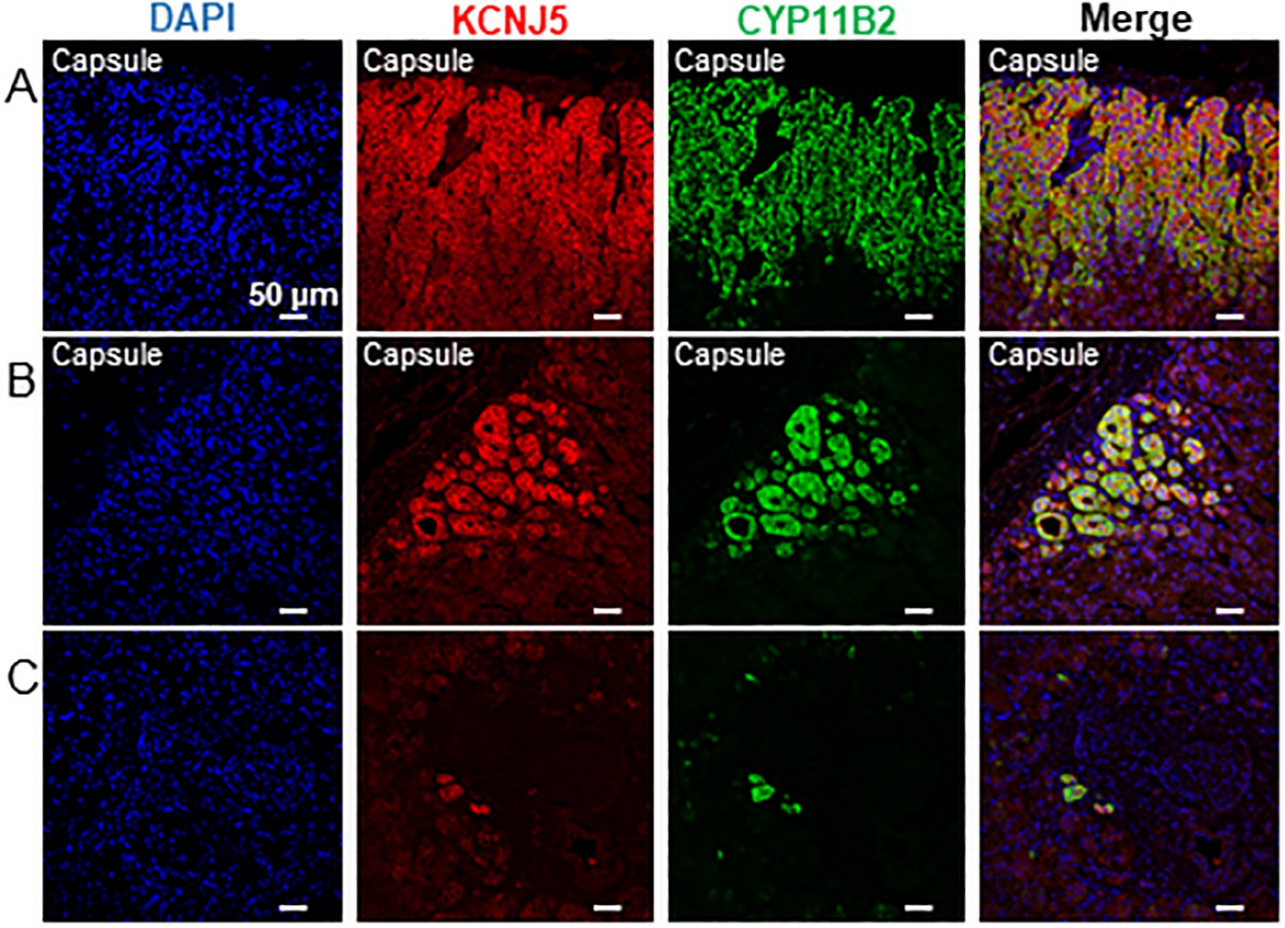
KCNJ5 expression in the normal human adrenal cortex. Double immunofluorescence of KCNJ5 and CYP11B2 reveals that KCNJ5 is highly expressed in aldosterone producing cells (A, B, C). Intense KCNJ5 immunolabelling is observed in CYP11B2 cells arranged in large (A) or small (B) APCC or isolated in the deep cortex (C). Cell nuclei were visualised with DAPI. (A) 42-year-old man. (B) 45-year-old man. (C) 52-year-old woman.

The G-protein-activated inward rectifying potassium channel Kir3.4 (also called KCNJ5 or GIRK4) is expressed in the ZG [17] and participates in the regulation of aldosterone production [18]. Double KCNJ5-CYP11B2 immunofluorescence showed co-localization of both proteins in all adrenals examined (Fig 7A–7C). It is interesting to notice that such co-localization was also present in the CYP11B2-positive cells located deep into the cortex (Fig 7C). Significant staining with KCNJ5 antibody was observed at the membrane of all cells belonging to the functional ZG. Moreover, a pronounced decrease in KCNJ5 immunostaining was evident in CYP11B2-negative cells relative to CYP11B2 positive cells (Fig 7A and 7B). We also found that all APMs identified in the adrenal cortex displayed strong KCNJ5 staining (Fig 7B).

S5 Fig. shows immunohistochemistry of Dab2, CYP11B2, and KCNJ5 on 3 consecutive whole adrenal sections. The images show that the immunoreactive surfaces for CYP11B2 and KCNJ5 are superimposed throughout the adrenal gland and similarly the subcapsular regions lacking immunoreactivity for CYP11B2 are also characterized by an absence of KCNJ5 expression (S5 Fig). In contrast, cells immunoreactive for Dab2 are distributed throughout the cortex without an expression restricted to cells expressing CYP11B2 (S5 Fig).

**Fig 8:**
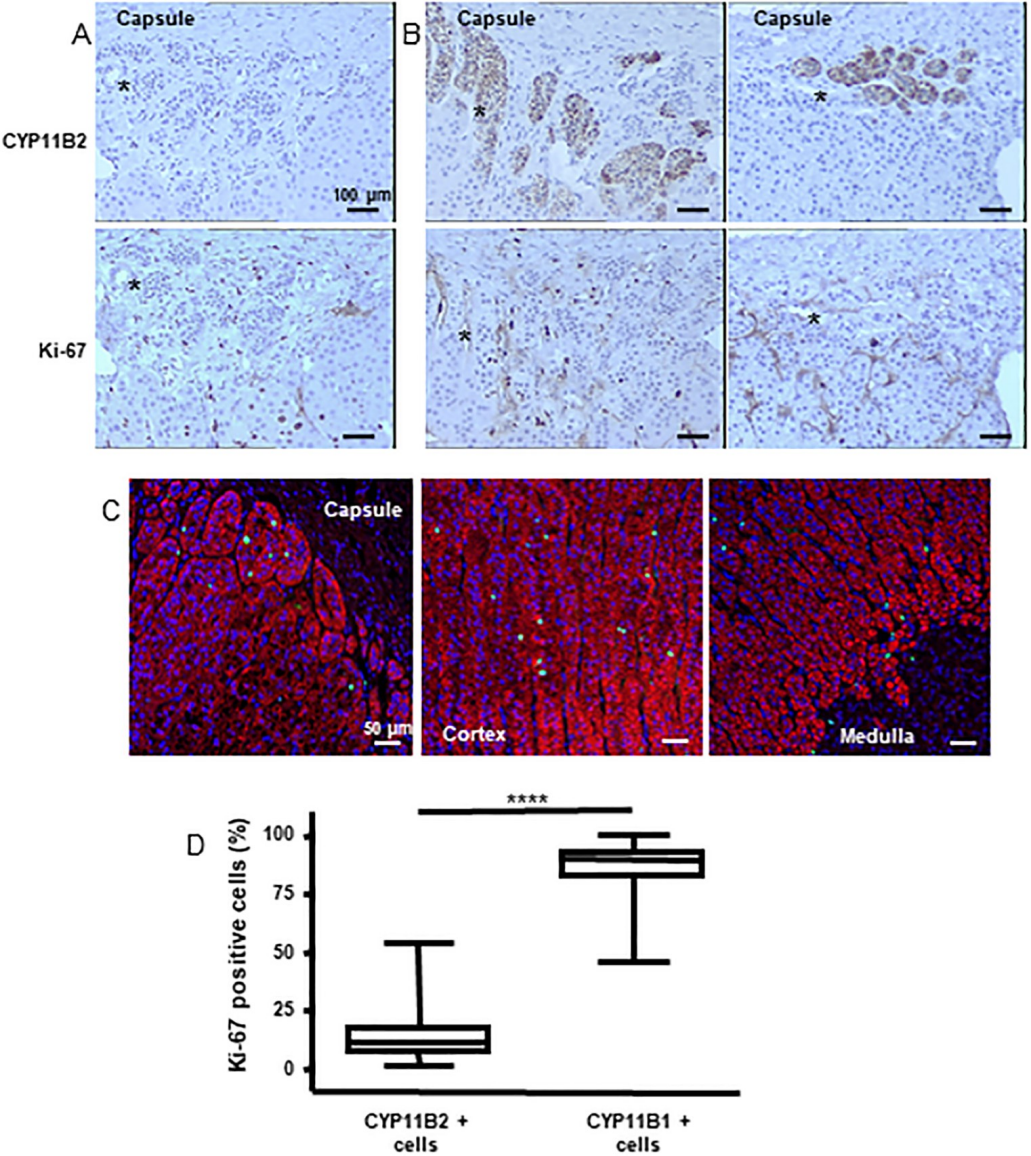
Proliferation occurs throughout all zones of the cortex irrespective of the functional status of the steroidogenic cells. (A, B) illustrative microphotographs showing the distributions of CYP11B2 and Ki-67 immunostainings in consecutives sections of two human adrenal cortices ((A) 68-year-old man. (B) 70-year-old woman). * denotes common structures on consecutive sections. (C) Double immunofluorescence for CYP11B1 (red) and Ki-67 (green) in the adrenal cortex of a 34-year-old man. Cell nuclei were visualised with DAPI (blue). (D) Box and whisker plot of Ki-67 percentage immunopositivity showing higher value associated with CYP11B1 expressing cells (**** P<0.0001).

Proliferation in normal human adrenal cortex has been uncovered by MIB-1 antibody, which recognizes Ki-67 nuclear antigen expressed in all phases of the cell cycle but not in resting cells [19]. We have detected Ki-67 immunoreactivity in all tissues examined with a high variability. Proliferation was not restricted either to a particular zone or a particular cellular phenotype as shown by immunochemistry on consecutive sections for Ki-67 and CYP11B2 or by double Ki-67-CYP11B1 immunofluorescence (Fig 8A–8C). Using consecutive sections, we demonstrated that Ki-67 expression occurred in 14.04% (±1.69%) of the CYP11B2-expressing cells while by double IF, Ki-67 appeared expressed in 85.96% (±1.69%) of the CYP11B1-positive cells (p<0.001, Fig 8D). Moreover, no significant correlation between Ki-67 and age or sex has been found in our cohort (S6A and S6B Fig).

**Fig 9:**
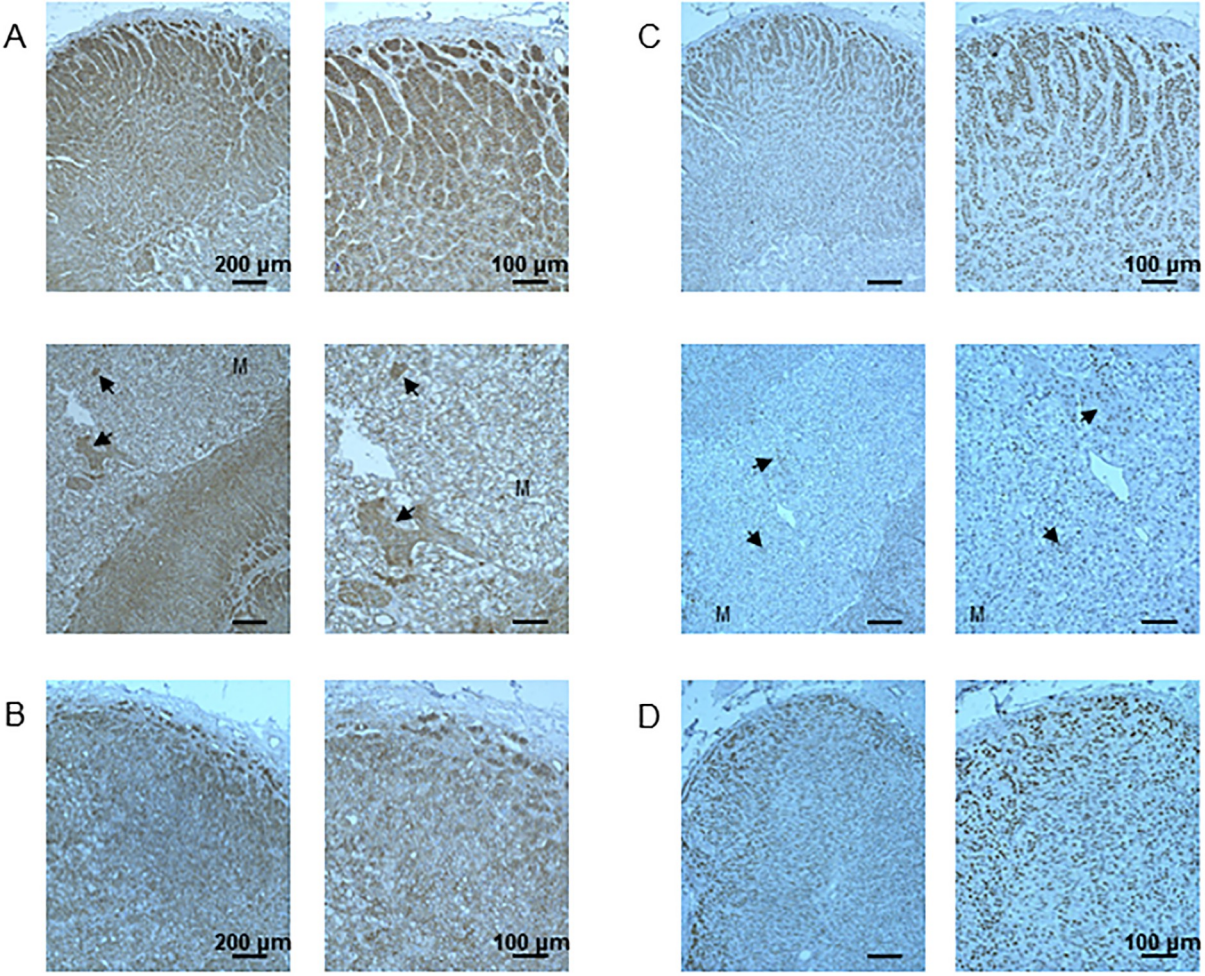
Wnt/β-catenin pathway is activated in normal human adrenal gland. Immunostaining for β-catenin (A and B) allows detection of the protein throughout the cortex at the membrane and cytoplasmic/nucleus levels while β-catenin expression in chromaffin cells of the medulla (M) is restricted to the membrane (A, lower microphotographs). Lef-1 expression is detected in the nucleus of cortical cells (C and D) and not in chromaffin cells (C, lower microphotographs). 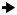 indicates cluster of cortical cells expressing β-catenin (A) or Lef-1 (C) in the medulla and in the vicinity of the central vasculature of the adrenal gland. (A and C) 30-year-old man. (B and D) 70-year-old man.

To determine whether β-catenin expression might represent a specific molecular signature for the ZG as previously shown in mice [20–22], we investigated its expression in our cohort of tissues. β-catenin plays a pivotal role in Wnt signal transduction in addition to its function as a cell-cell adhesion component. When activated by Wnt ligands, β-catenin accumulates in the cytoplasm and then translocates into the nucleus, where it interacts with the transcription factors TCF/Lef-1, and activates downstream target genes [23]. We observed that β-catenin protein was detected in the whole cortex as well as in the medulla regardless of donor age or sex (Fig 9A and 9B). The subcellular localization was membranous as well as cytoplasmic and nuclear (Fig 9A and 9B), an observation which might be reflect an activation of the pathway in cortical cells. In contrast, β-catenin expression was exclusively restricted to the membrane in the chromaffin cells of the medulla (Fig 9A). Interestingly, these two different patterns of β-catenin expression allow discrimination of cortical cells from chromaffin cells throughout the medulla (Fig 9A).

To address the biological relevance of Wnt/β-catenin pathway activation, we evaluated the nuclear protein expression of Lef-1, a Wnt/β-catenin target gene [24, 25]. Lef-1 is expressed throughout the cortex (Fig 9C and 9D) overlapping the β-catenin expression. Interestingly, Lef-1 expression is also observed in cortical cells found in the medulla (Fig 9C). Consistent with the non-activated state of the Wnt/β-catenin pathway in the medulla, a complete lack of Lef-1 immunoreactivity was observed in the chromaffin cells (Fig 9C).

## Discussion

In line with previous reports [10, 26, 27], the present study confirms that the functional ZG and ZF delineated through immunohistochemical approaches do not overlap the histological zonation of the human adrenal cortex established through H&E staining. Thus, we describe in our entire cohort a loss of zonation and intermingling of cells that express CYP11B1 and CYP11B2 throughout the cortex and the medulla, further demonstrating the absence of clear functional zonation. Altogether, these observations clearly indicate that the function only partially follows morphology/localization patterns. In rodents, two main models have been put forward to explain adrenocortical zonation: (i) the centripetal migration model [28] and (ii) the zonal model [29]. In the centripetal migration model, undifferentiated progenitor cells in the capsule or subcapsular region give rise to terminally differentiated mineralocorticoid-producing ZG cells. These cells migrate inwardly and undergo lineage conversion into glucocorticoid-producing ZF cells before undergoing apoptosis at the corticomedullary junction [30]. In contrast, the zonal model states that each zone develops and is maintained independently by zone-specific progenitor cells with sharp boundaries between different cell types. Lineage tracing studies have demonstrated that cells located in the subcapsular and capsular regions have the same properties as stem/progenitor cells which upon differentiation and centripetal migration populate the entire cortex [31–33], providing evidence for the migration model in rodents. The mechanism involved in the maintenance of the differentiated cortex probably follows different ways in these species by activating specific signaling pathways besides those targeted by trophic factors such as angiotensin II and potassium for the ZG cells and ACTH for the ZF cells. Our data together with previously published results [11, 12, 26, 27], suggest that the model of functional zonation defined in rats and mice (two animal models used as surrogates for the human adrenal cortex) may not be extrapolated to humans and appears therefore inconclusive regarding the maintenance of zonal differentiation of the human cortex. In fact, while the functional ZG does correspond to the H-E defined zonation for individuals between birth and 11 years of age [11, 12, 26, 27], the adrenal cortex then rapidly undergoes profound functional remodeling during life span. This process likely results from various causes such as the high sodium/low-potassium diet which induces a suppression of the renin-angiotensin-aldosterone system and subsequently a lack of stimulation of the ZG [11, 12, 26, 27]. As a consequence, the cells expressing CYP11B2 progressively begin to decline in the subcapsular region and are replaced by neighboring CYP11B1-expressing cells probably under the influence of the extracellular matrix (ECM) scaffold which contains binding domains and releases chemoattractant molecules [34]. Moreover, we hypothesize that, since the progressive disappearance of the functional ZG is observed years before the detection of APMs, the development of APMs might represent a compensatory overgrowth of the remaining CYP11B2 cells in order to fulfill the organism’s requirement for aldosterone production. Another possibility would involve the occurrence of aldosterone-driving mutations in some ZG cells giving rise thereafter to APMs, in agreement with the detection of somatic mutations of aldosterone synthesis driver genes in APMs in normal adrenal glands [35, 36]. In addition, because most of the donors have died from acute illnesses which are known complications of hypertension, we cannot rule out that, in some subjects, a history of hypertension may have influenced the *CYP11B2* expression pattern. In mice, ZG was also shown to exhibit some discontinuity in the CYP11B2 expressing cell sheets however, no CYP11B1 cells have been identified beneath the capsule [37, 38]. It is conceivable that the discontinuity of the functional ZG is a consequence of the presence of progenitor cells known to be negative for CYP11B2 and to reside in the ZG [33]. Furthermore, it has been shown that a high salt diet in mice could cause functional disorganization of the adrenal cortex [39]. However, the current agreement suggests that the parenchymal cells of the adrenal cortex represent a single cell type which successively differentiates into three different functional states, *i*.*e*. the mineralocorticoid-, glucocorticoid-, or androgen-secreting phenotypes, throughout its centripetal migration from the capsule to the corticomedullary junction. Cell-lineage tracing definitively demonstrated that progenitor/stem cells in the outer portion of the gland contribute to the differentiated adrenocortical cells which in turn participate to populate the inner zones of the cortex [33, 40]. Naturally, such evidences are impossible to collect in humans however, analysis of our data might suggest an independent model besides the lineage conversion on the basis of the following findings: (i) the maintenance of cell phenotype does not depend on cell location within the gland; (ii) the progressive disappearance and replacement of CYP11B2-expressing cells by CYP11B1-expressing cells; (iii) the occurrence of CYP11B2 cells in the inner cortex and of both cell types in the medulla; and (iv) expression of proliferation markers by steroidogenic cells throughout the cortex instead of its restriction to the outermost layers. In fact, the latter observation suggests that the maintenance of tissue homeostasis may not involve a population of stem/progenitor cells, but may be ensured by differentiated steroidogenic cells. As such, the human adrenal cortex demonstrates a previously unanticipated remodeling capability occurring during the entire life span through inward displacement of terminally differentiated cortical cells towards the central vasculature. Interestingly, morphology and spatial organization of CYP11B2 expressing cells found in the deep cortex were indistinguishable from those of the neighboring CYP11B1-expressing cells. Likewise, the CYP11B1 cells found beneath the capsule displayed a structural arrangement identical to that of the histological ZG. We might conclude that the organization of tissue areas primarily depends on the cortical sublocalization of cells rather than by its function.

In the absence of reliable antibodies, RT-PCR experiments on glomerulosa cells [41] and *in situ* hybridization studies of human adrenal cortex [42–44] have first been carried out to study expression of mRNAs encoding steroidogenic enzymes. CYP11B1 mRNAs were detected in cultured glomerulosa cells [41], a finding which is in agreement with our results showing the presence of CYP11B1-expressing cells beneath the capsule. Moreover, data from Pascoe et al [43] showed that CYP11B1 expression was detectable in the ZG of normal adrenal sections while CYP11B2 mRNA was found to be strongly expressed in the same region although not in all samples studied. On the other hand, Enberg et al. [42] reported that expression of CYP11B2 was very low in two out of the five normal adrenals studied, an observation which may result from the discontinuous expression of CYP11B2 protein.

The two histological components of the adrenal gland, namely the cortex and the medulla, have long been considered as physically separated, despite the fact that no capsule was identified at the corticomedullary junction. In this study, we observe that the intermingling between both tissues exists to a large extent, confirming previous data obtained in a small cohort of normal adrenals [14]. Our data show that both types of functional adrenocortical cells are present in the medulla suggesting that chromaffin cell secretory products can influence the production of corticosteroids, including not only cortisol but also aldosterone, through a paracrine mode of communication.

Dab2 is a mitogen-responsive phosphoprotein that modulates the growth factor/Ras pathways. In this respect, it is noteworthy that the IGF-receptor is widely expressed in all three zones of the adrenal cortex [45]. Thus, Dab2 expression at the cytoplasmic membrane detected here suggests its possible involvement in regulating this pathway and might not be correlated to a particular steroidogenic phenotype or functional zone [16, 17].

The human adrenal cortex expresses high levels of KCNJ5 in CYP11B2 positive cells independently of their location and age of the donor. *KCNJ5* mutations are absent in functional ZG as well as in APMs of normal adrenal cortex [35, 36]. Only 34% of the APMs studied were identified with mutations in other ion channels/pumps genes such as *ATPase*, *Na+/K+ transporting*, *α1-polypeptide* (*ATP1A1*) and *calcium channel*, *voltage-dependent*, *L-type*, *α1D-subunit* (*CACNA1D*), in contrast to the overall prevalence of 43% of *KCNJ5* mutations found in Aldosterone Producing Adenomas (APAs, [46]). These discrepancies suggest that the APAs may not directly result from APMs and it is conceivable that both tissues are separate entities although a recent study has shown that *KCNJ5* mutations might be found in APMs from adrenals with APA [47].

In mice, β-catenin has been shown to be preferentially expressed in the ZG with a localization pattern including the cytoplasmic membrane and the nucleus, suggesting an active canonical Wnt/β-catenin pathway [20, 21, 38, 48]. The absence of β-catenin expression in the inner cortex of the mouse adrenal gland is not completely understood since β-catenin also fulfills a structural role in adhesion junctions where, together with cytosolic α-catenin, it forms a complex that links membrane cadherins to actin cytoskeleton [49]. It has been proposed that the restricted expression of β-catenin is related to the presence of stem/progenitor cells in the subcapsular region of the gland [37]. In humans, β-catenin protein expression was found either exclusively in ZG [50] or in the entire cortex [51]. Our data are rather in accordance with the latter. However, for both studies [50, 51] activation of β-catenin, as shown by cytoplasmic and nuclear accumulation, was restricted to the histological ZG whereas our data, based on the expression of the β-catenin target gene Lef-1, suggests that the pathway is also activated in the inner zones [52]. Rege et al. [53] have previously reported that Lef-1 is expressed in the outer part of the cortex but not in the inner region of the tissue. This discrepancy might reside in the lack of specificity and sensitivity of the antibody and possibly in the different protocols used for tissue fixation and epitope unmasking.

Sasano et al [54] have shown that cell proliferation in human adrenals occurs predominantly in the outer ZF based on histological recognition of the cells. Our data indicate, by using the newly generated antibodies against CYP11B1 and CYP11B2 combined with Ki-67 expression that proliferation rather occurs throughout the cortex with a strong prevalence in CYP11B1-expressing cells.

In summary, using immunohistochemical analyses of the assignment of steroidogenic enzymes to ZG or ZF cells in the normal human adrenal cortex, our study shows: i) a loss of zonation and intermingling of cells that express CYP11B1 and CYP11B2 throughout both the cortex and the medulla; and ii) finally, that the functional status is the unique way to impute a cell to a zone. Our data cannot exclude the existence of the CYP11B2+ cell conversion/migration lineage as demonstrated in mice but suggest for the first time that an alternative mechanism may exist in the human adrenal such as inward displacement without lineage conversion.

## Supporting information

S1 Table Sexe, age and cause of death of kidney transplant donors. (DOCX)

S1 Fig Immunohistochemistry of CYP11B2 protein expression in human adrenals during aging. (A) 22-year-old man. (B) 31-year-old man. (C) 45-year-old woman. (D) 52-year-old woman. (E) 63-year-old man. (F) 70-year-old woman. Each panel shows two different areas of the same adrenal at two magnifications. CYP11B2 expression is always discontinuous in our cohort. Lower image of each panel depicts small groups of cells and/or single cell positive for CYP11B2. A progressive thickening of the area positive for CYP11B2 is observed in D-F. Large APMs in the subcapsular area are identified in E and F (▸). (G) Correlation between CYP11B2 positive surface normalized to adrenal cortex surface (%) and aging. r: Pearson correlation.
(TIF)

S2 Fig Representative images used for quantification analysis. A and D, Original low-magnification scanned images of adrenal with SF1 and CYP11B2 immunohistochemistry, respectively. B and D, Analysis of adrenal cortex areas using threshold tool of ImageJ for each image. C and F, Analysis of SF1 and CYP11B2 expressing areas using threshold tool of image, respectively. The percentage of CYP11B2 positive area relative to adrenal cortex were then calculated.
(TIF)

S3 Fig Triple immunofluorescence for CYP11B1, CYP11B2 and SF-1. All cells positive for the steroidogenic cell marker SF-1 were expressing either CYP11B1 or CYP11B2 in the adrenal cortex of a 68-year-old woman. Corticosteroid producing cells were neither negative nor positive for both enzymes. Triple immunofluorescence for CYP11B1 (red), CYP11B2 (green) and SF-1 (magenta).
(TIF)

S4 Fig Immunohistochemmistry for CYP11B2 and immunofluorescence for CYP11B1 and CYP11B2 on consecutive sections of an adrenal gland of a 45 years-old female donor. Section 0 identified a group of cells positive for CYP11B2. Fourteen consecutive sections were cut. CYP11B2 labeling was performed on sections 5 and 10 to allow global identification of the tissue. On sections 1, 4, 7, 8, 9 11 and 14 an immunofluorescent labelling for CYP11B1 and CYP11B2 allowed to highlight the intermingling of these cells in the depth of the adrenal cortex. * identifies common structure on consecutive sections.
(TIF)

S5 Fig Immunohistochemistry for Dab2, CYP11B2 and KCNJ5. Three consecutive 5μm paraffin sections were cut from a block of an adrenal gland from a 45year-old male donor. All sections were counterstained with hematoxylin. Whole adrenal images show an overlay of CYP11B2 and KCNJ5 labeling while Dab2 labeling is present throughout the cortex.
(TIF)

S6 Fig Proliferation in normal human adrenal cortices. Associations between percentage of Ki-67 positive cells and (A) sex and (B) age in CYP11B2 and CYP11B1 compartments, are shown in 47 normal adrenals. Open circles represent the CYP11B2 positive cells and close circle represent CYP11B1 positive cells. Abbreviations: W, women; M, men; ns, not significant. Data were analyzed using (A) Mann-Whitney *U* test and (B) Pearson correlation test. r: Pearson correlation.
(TIF)
