# Supplementary material for: Ectopic localization of CYP11B1 and CYP11B2-expressing cells in the normal human adrenal gland
Source: PLoS One. 2022 Dec 30;17(12):e0279682. doi: 10.1371/journal.pone.0279682 (PMC9803228; doi:10.1371/journal.pone.0279682)
Supplement: S1 Table — (DOCX) [file pone.0279682.s001.docx]

**S1 Tab. Sexe, age and cause of death of kidney transplant donors.**

**Female No. Age (years) Cause of death Male No. Age (years) Cause of death**

1 34 Heart failure 1 22 Heart failure

2 39 Heart failure 2 27 Head trauma

3 45 Heart failure 3 29 Heart failure

4 45 Heart failure 4 30 Heart failure

5 49 Aneurysm rupture 5 31 Gun shot wound

6 52 Aneurysm rupture 6 42 Head trauma

7 53 Aneurysm rupture 7 43 Heart failure

8 62 Stroke 8 44 Aneurysm rupture

9 64 Aneurysm rupture 9 44 Heart Failure

10 64 Aneurysm rupture 10 45 Heart failure

11 64 Stroke 11 45 Stroke

12 67 Stroke 12 45 Heart failure

13 68 Stroke 13 45 Heart failure

14 70 Stroke 14 46 Heart failure

15 71 Head trauma 15 47 Head trauma

16 73 Stroke 16 49 Stroke

17 77 Stroke 17 52 Aneurysm rupture

18 78 Head trauma 18 53 Aneurysm rupture

19 57 Heart failure

20 60 Aneurysm rupture

21 60 Heart failure

22 62 Stroke

23 63 Heart failure

24 66 Stroke

25 66 Stroke

26 70 Head trauma

27 70 Stroke

28 71 Head trauma

29 81 Head trauma
