# Supplementary figures and images for: Ectopic localization of CYP11B1 and CYP11B2-expressing cells in the normal human adrenal gland
Source: PLoS One. 2022 Dec 30;17(12):e0279682. doi: 10.1371/journal.pone.0279682 (PMC9803228; doi:10.1371/journal.pone.0279682)

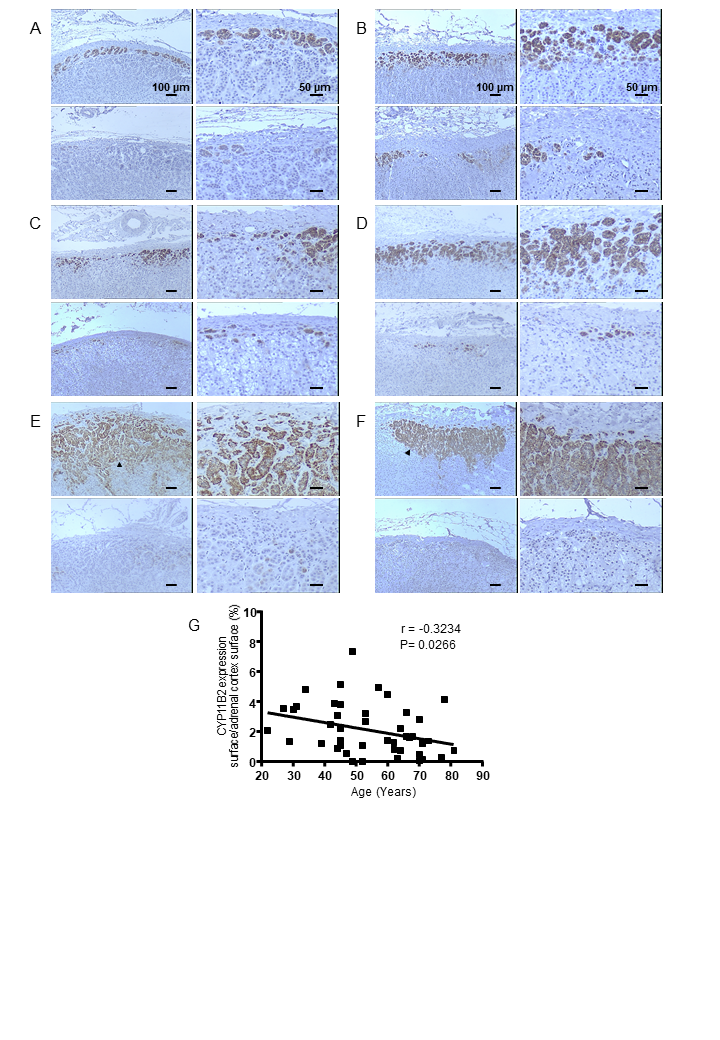

Supplement: S1 Fig — (A) 22-year-old man. (B) 31-year-old man. (C) 45-year-old woman. (D) 52-year-old woman. (E) 63-year-old man. (F) 70-year-old woman. Each panel shows two different areas of the same adrenal at two magnifications. CYP11B2 expression is always discontinuous in our cohort. Lower image of each panel depicts small groups of cells and/or single cell positive for CYP11B2. A progressive thickening of the area positive for CYP11B2 is observed in D-F. Large APMs in the subcapsular area are identified in E and F (▸). (G) Correlation between CYP11B2 positive surface normalized to adrenal cortex surface (%) and aging. r: Pearson correlation. (TIF) [file pone.0279682.s002.tif]

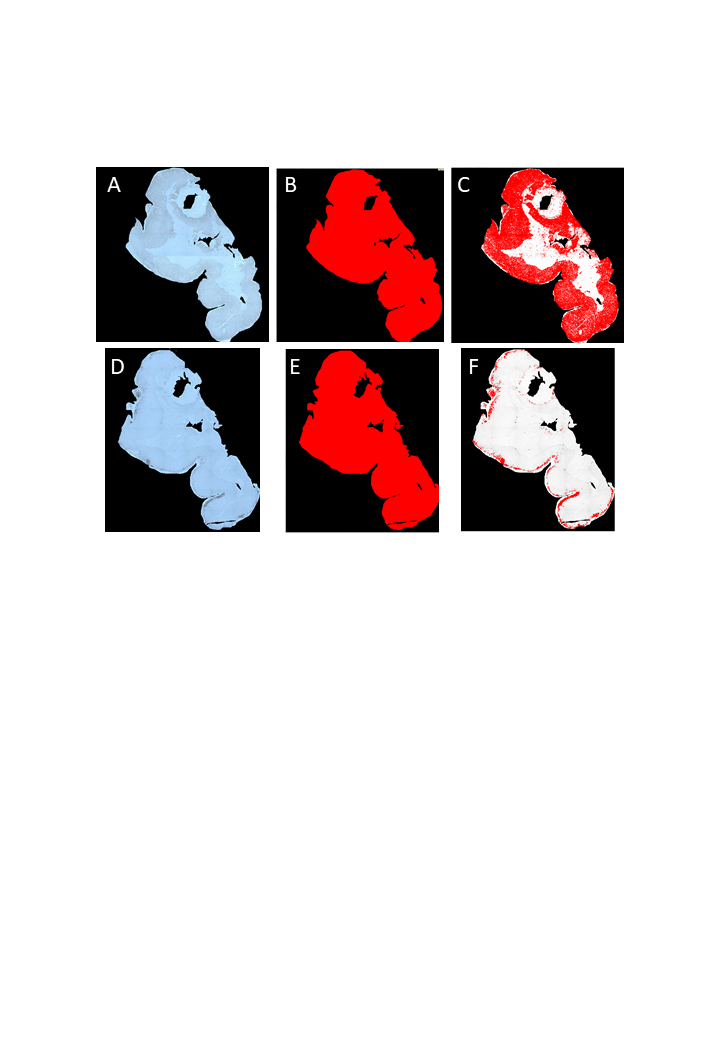

Supplement: S2 Fig — A and D, Original low-magnification scanned images of adrenal with SF1 and CYP11B2 immunohistochemistry, respectively. B and D, Analysis of adrenal cortex areas using threshold tool of ImageJ for each image. C and F, Analysis of SF1 and CYP11B2 expressing areas using threshold tool of image, respectively. The percentage of CYP11B2 positive area relative to adrenal cortex were then calculated. (TIF) [file pone.0279682.s003.tif]

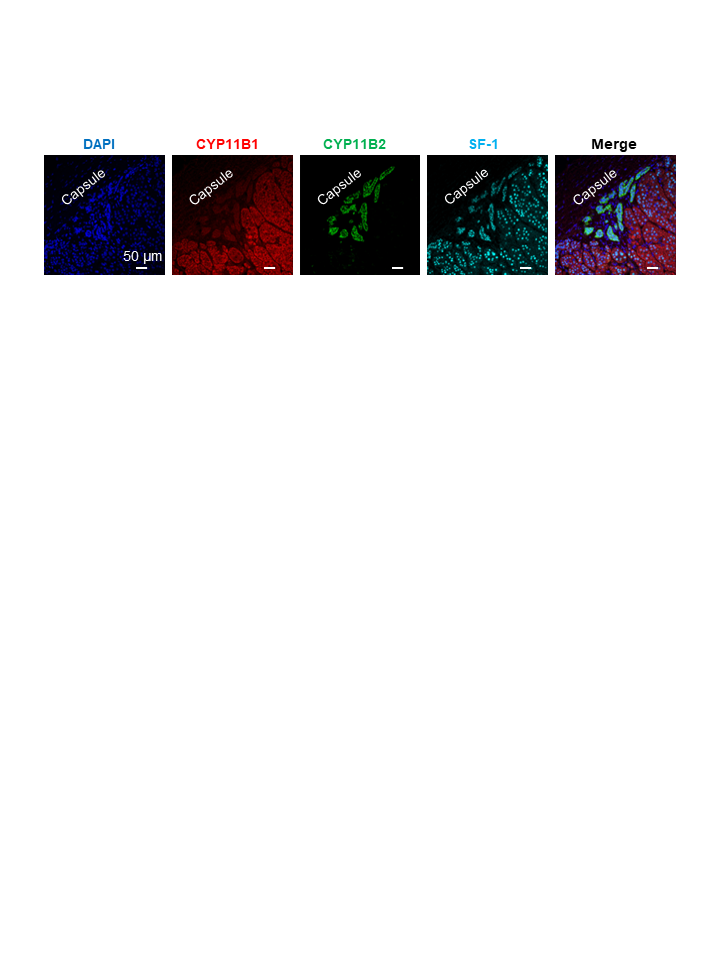

Supplement: S3 Fig — All cells positive for the steroidogenic cell marker SF-1 were expressing either CYP11B1 or CYP11B2 in the adrenal cortex of a 68-year-old woman. Corticosteroid producing cells were neither negative nor positive for both enzymes. Triple immunofluorescence for CYP11B1 (red), CYP11B2 (green) and SF-1 (magenta). (TIF) [file pone.0279682.s004.tif]

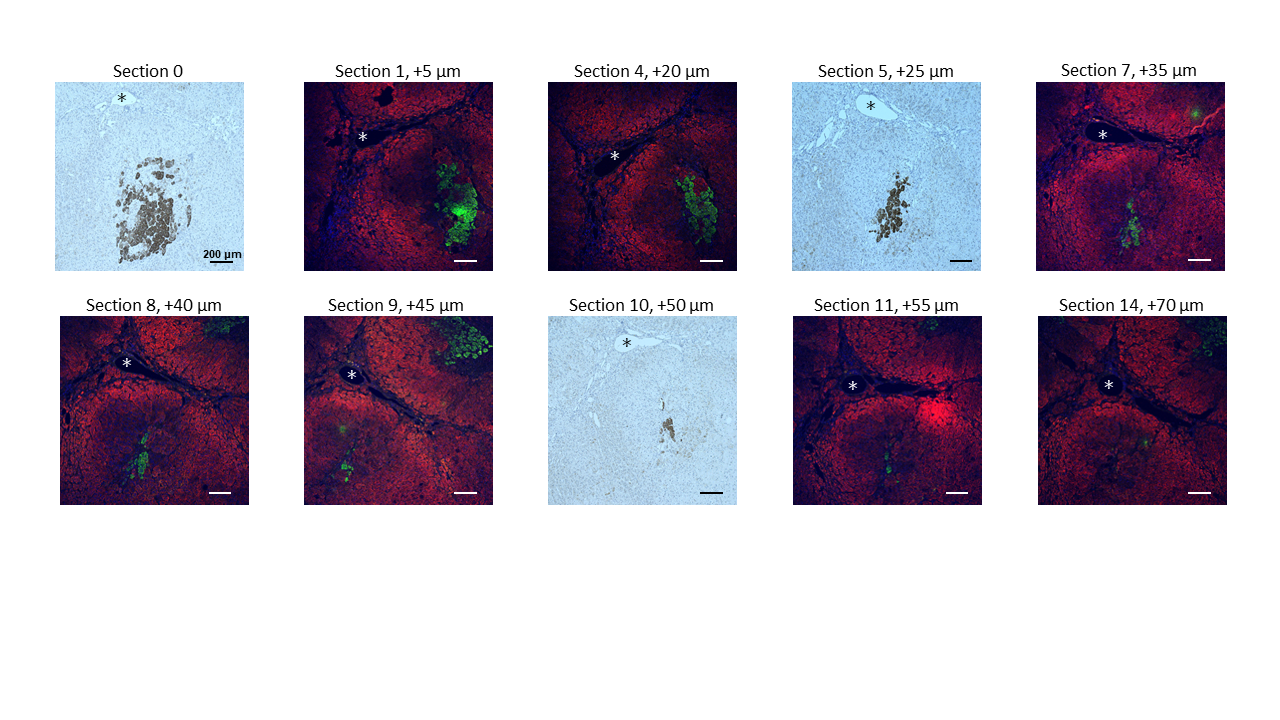

Supplement: S4 Fig — Section 0 identified a group of cells positive for CYP11B2. Fourteen consecutive sections were cut. CYP11B2 labeling was performed on sections 5 and 10 to allow global identification of the tissue. On sections 1, 4, 7, 8, 9 11 and 14 an immunofluorescent labelling for CYP11B1 and CYP11B2 allowed to highlight the intermingling of these cells in the depth of the adrenal cortex. * identifies common structure on consecutive sections. (TIF) [file pone.0279682.s005.tif]

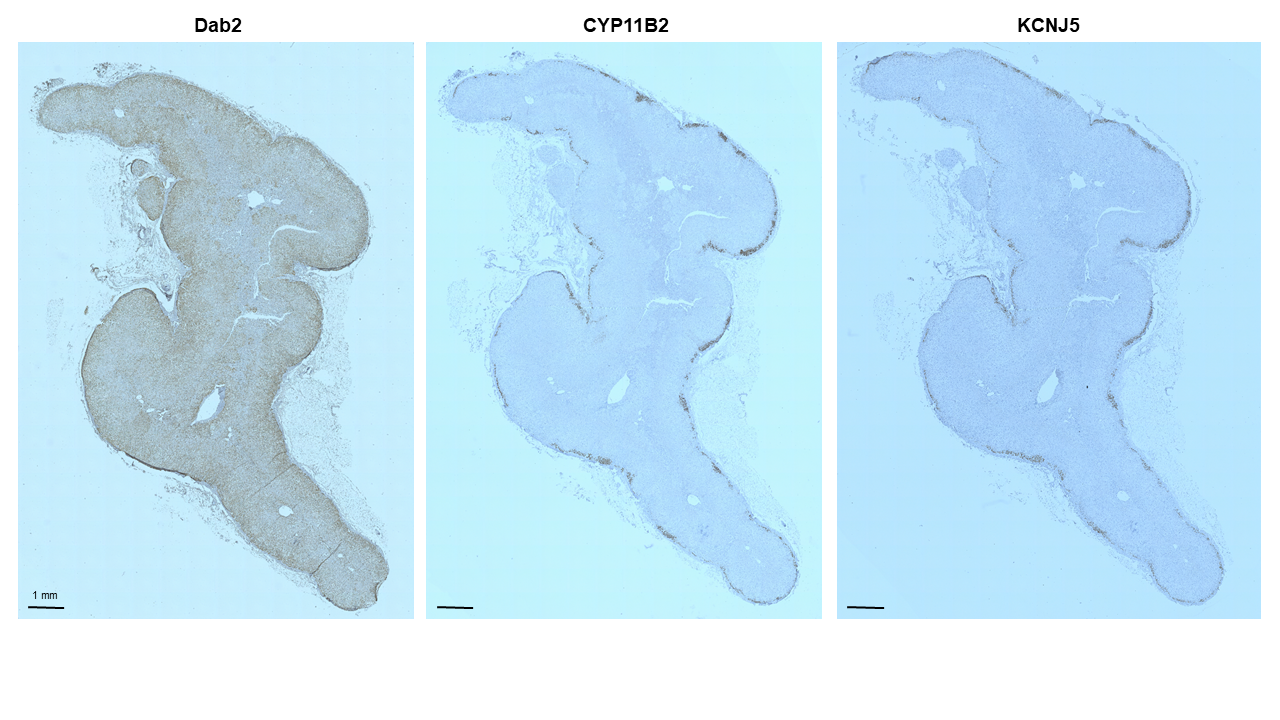

Supplement: S5 Fig — Three consecutive 5μm paraffin sections were cut from a block of an adrenal gland from a 45year-old male donor. All sections were counterstained with hematoxylin. Whole adrenal images show an overlay of CYP11B2 and KCNJ5 labeling while Dab2 labeling is present throughout the cortex. (TIF) [file pone.0279682.s006.tif]

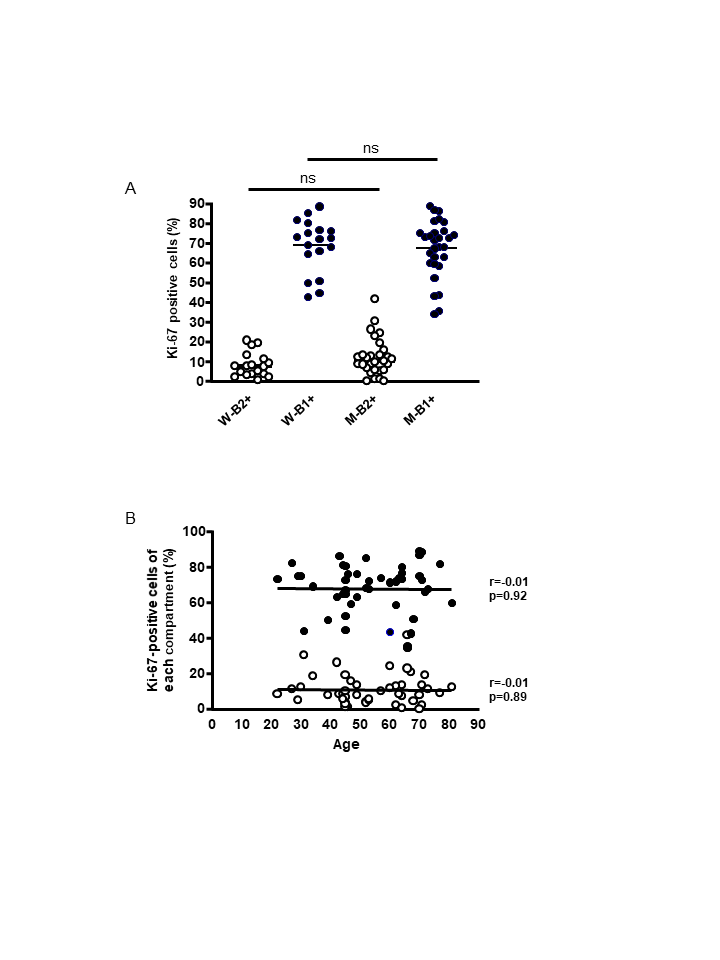

Supplement: S6 Fig — Associations between percentage of Ki-67 positive cells and (A) sex and (B) age in CYP11B2 and CYP11B1 compartments, are shown in 47 normal adrenals. Open circles represent the CYP11B2 positive cells and close circle represent CYP11B1 positive cells. Abbreviations: W, women; M, men; ns, not significant. Data were analyzed using (A) Mann-Whitney U test and (B) Pearson correlation test. r: Pearson correlation. (TIF) [file pone.0279682.s007.tif]
